# 2500-g Low Birth Weight Cutoff: History and Implications for Future Research and Policy

**DOI:** 10.1007/s10995-016-2131-9

**Published:** 2016-07-23

**Authors:** Michelle M. Hughes, Robert E. Black, Joanne Katz

**Affiliations:** Department of International Health, Johns Hopkins Bloomberg School of Public Health, 615 North Wolfe Street, Baltimore, MD 21205 USA

**Keywords:** Low birth weight, Preterm birth, Small for gestational age, Fetal growth, Intrauterine growth restriction

## Abstract

*Purpose* To research the origins of the 2500 g cutoff for low birth weight and the evolution of indicators to identify newborns at high mortality risk. *Description* Early research concluded “prematurity”, measured mainly through birth weight, was responsible for increased health risks. The World Health Organization’s original prematurity definition was birth weight ≤2500 g. 1960s research clarified the difference between gestational age and birth weight leading to questions of the causal role of birth weight for health outcomes. Focus turned to two etiologies of low birth weight, preterm births and intrauterine growth restriction, which were both causally associated with morbidity and mortality but through different pathways; a standard cutoff based on gestational age or customized cutoff was debated. *Assessment* While low birth weight can be due to preterm or intrauterine growth restriction (or both), the historic 2500 g cutoff remains the standard by which the majority of policy makers define low birth weight and use it to predict perinatal and infant adverse outcomes. *Conclusion* Current efforts to refocus research on preterm births and poor intrauterine growth are important to understanding the direct causes of mortality rather than low birth weight as a convenient surrogate. Such distinctions also allow researchers and practitioners to test and target interventions outcomes more effectively.

## Significance

*What is already known on this subject?* 2500 g is the standard cutoff used to identify low birth weight infants at high mortality risk.

*What this study adds?* This study provides the historical context for this low birthweight standard and more recent trends to link early morbidity and mortality to their etiological causes of birth size.

## Purpose

Public health and medical professionals have categorized infants as low birth weight for almost a century. These infants have 20 times greater likelihood of dying compared to infants of normal birth weight (McCormick 1985; Wilcox 2001). Birth weight is a measure that is easily accessible through vital records compared to gestational age, especially in low resource settings. Whether birth weight, independent of preterm and poor fetal growth, is in the causal pathway for mortality is still debated. Birth weight may serve as a convenient surrogate for other factors causally associated with mortality and other adverse outcomes. The current reduction in the global prevalence of low birth weight is a goal set by the World Health Assembly as a nutrition indicator. This illustrates the persistence of this indicator and the confusion of interpretation it produces. This paper traces the origins of the low birth weight indicator through current thinking on its uses in global health research, policy and programs.

## Assessment

### Historical Origins

Early twentieth century papers provided generalizations of the characteristics of premature infants rather than gestational age categories. A study in 1923 described the criteria for establishing prematurity as “weight, length, and general characteristics, such as facies, texture of the skin, undeveloped nails, cry, unstable temperature and history of expected birth” (Talbot et al. 1923). At this time, factors beyond gestational age were considered to contribute to the definition of prematurity, including birth weight.

A 1902 paper described a premature infant as having a weight that “varies from 3 to 4 1/2 lb” (Ballantyne 1902). The author commented that anecdotally morbidity is close to 100 % and mortality 50 % for premature infants. A 1906 textbook on the feeding and hygiene of premature and full term infants gives mortality data categorized by weight from a French hospital in 1898 (Budin 1907). In the department for “weaklings”, the following mortality by weight: (1) <1200 g—95 %, (2) 1200–1499—85 %, (3) 1500–1999—61 %, and (4) >2000—33 % was presented. A 1915 review of 10,000 births in New York City categorized infants as premature if they were born either “before term” (noting the difficulty of ascertaining gestational age), measured <46 cm, or weighed <2275 g (Holt and Babbit 1915). Prematurity was considered the largest contributor to the observed neonatal mortality of 3.1 %. Optiz termed children born <2750 g as underweight in 1914 (Optiz 1914; Pearce 1919). In 1918 Taylor published data on treatment of prematurity using a sample of 60 premature infants, whose weights ranged from 1505 to 2860 g; a case definition of premature was not provided (Taylor 1918).

Dr. Arvo Ylppö, a Finnish pediatrician, first proposed the 2500 g cutoff in 1919 (Ylppö 1919). He described a cohort of 2168 infants born in a German health facility between 1909 and 1918. Of these infants, 5.3 % were born preterm. While he provided no justification for this specific weight cutoff, it has become the global marker for low birth weight.

Other researchers began to adopt this cutoff in their analyses, although not uniformly. In 1920 Schwarz and Kohn analyzed low birth weight stating “we have arbitrarily made 2500 g or under, the weight designated as low birth weight” and found that low birth weight infants had ten times the mortality of normal weight infants in the first month (Schwarz and Kohn 1921). A 1923 study on prematurity used a series of 20 infants with the largest infant weighing only 2098 g (Talbot et al. 1923) whereas a 1927 study included weights up to 3000 g (Hess and Chamberlin 1927). Capper’s (1928) study used 2500 g but then concluded only 72 % of those <2500 g were premature, acknowledging a demarcation between the definition of low birth weight and prematurity (Capper 1928). The heaviest infant in a 1931 study of respiration in premature infants was 2296 g with no definition of criteria for inclusion as “premature” (Shaw and Hopkins 1931). Clifford published two papers on premature infants in 1934 with a weight criterion of 2270 g or less and observed 38 % mortality in low birth weight compared to 1 % in those who were above 2270 g (Clifford 1934a, b).

As there was no universal standard, it was difficult to compare morbidity and mortality outcomes of low birth weight infants between studies. Dr. Ethel Dunham, during the 5th annual meeting of the American Academy of Pediatrics (AAP) in 1935, summarized the problem as follows:

Reports from hospitals of mortality from prematurity also differ widely. The differences may be attributed in part to variability in criteria for the diagnosis of prematurity, in part to differences in the periods of observation at the end of which the report is made, and in part to the numbers of infants in high or low weight groups. For example, Clifford reports as premature only those infants who weight five pounds or less at birth, while Hadley used the criteria established by Ylppö, namely, a birth weight of 2500 g or less. Other observers base the diagnosis on other criteria such as history of period gestation, clinical evidences or length, etc. As far as the period of observation is concerned, it is obvious that the same periods as well as the same criteria for diagnosis must be used by different observers to make results comparable.

Later in the meeting a standard criterion of prematurity (≤2500 g regardless of period of gestation) to facilitate comparison across studies was proposed. This meeting codified the 2500 g division first proposed by Ylppö in 1919.

Subsequent papers began to use definitions in publications with some exceptions. A 1938 review of over 15,000 deliveries focused on the attributes of premature births (Anderson and Lyon 1939). Peckham used the 2500 g cutoff and a length of 45 cm (Peckham 1938). A 1941–1945 US cohort of premature infants used a 2250 g cut-off to examine mortality, stratified by gestational age (Steiner and Pomerance 1950). In a 1948 JAMA paper, prematurity was defined as “any infant born alive who weighs 2500 g (5 pounds, 8 ounces) or less…This definition is that which is accepted by the AAP and must be rigidly adhered to by all institutions and agencies for reports and tabulations if experiences are to be comparable” (Koch et al. 1948). The World Health Organization (WHO) Expert Group on Prematurity proposed that the nebulous international definition of prematurity be a liveborn infant ≤2500 g or specified as “immature” or born <37 weeks gestation (Expert Group on Prematurity 1950). This recommendation was adopted by the First World Health Assembly in 1948 as the global standard.

### Incorporation of Gestational Age

In the 1950s there was a growing awareness of the limitations of using birth weight as the definition of prematurity. Studies began to use gestational age and birth weight to examine neonatal mortality (Steiner and Pomerance 1950) and differences in underlying etiologies between low birth weight and preterm births (Record et al. 1952). In 1955 Schlesinger and Allaway explicitly stated that birth weight was a poor indicator of prematurity, even though it was easier to measure than gestational age (Schlesinger and Allaway 1955). They presented data from New York City showing longer gestation was associated with decreased mortality within low birth weight categories (<2500 g); a similar pattern was observed within gestational age groups (<36 weeks) for birth weight.

As the quality of data on gestational age improved, many studies were published on the distributions of birth weight and gestational age and their relationships to neonatal morbidity and mortality (Battaglia et al. 1966; Brimblecombe and Ashford 1968; Erhardt et al. 1964; Karn and Penrose 1951; Lubchenco et al. 1963, 1972; Puffer and Serrano 1973; Taback 1951; Williams et al. 1982). Infants of lower birth weight and earlier gestational age were at highest risk for neonatal morbidity and mortality.

At the same time, a more nuanced understanding of the differences between low birth weight, premature, and small for gestational age (SGA) emerged (Battaglia et al. 1966; Gruenwald 1964; Lubchenco 1976; Silverman et al. 1967; van den Berg and Yerushalmy 1966; Wilcox 2001). In 1966, the AAP proposed nomenclature to characterize intrauterine growth (small, appropriate, and large-for-gestational age) although declined to provide numeric cutoffs, citing the need for more data (Battaglia and Lubchenco 1967; Silverman et al. 1967).

Concurrently, a universal birth weight cutoff was now thought not to be appropriate as certain population characteristics modified the relationship between birth weight and adverse birth outcomes (Gruenwald 1964). These factors included ethnicity, altitude, geographic setting (urban versus rural), sex, parity, age, nutrition, smoking and socioeconomic status (Brimblecombe and Ashford 1968; Chase 1969; Committee to Study the Prevention of Low Birthweight & Prevention 1985; Erhardt et al. 1964; Karn and Penrose 1951; Kramer 1987; Lubchenco et al. 1963; Pethybridge et al. 1974; Rooth 1980; Sansing and Chinnici 1976; Saugstad 1981). The distribution of birth weights was shifted based on these factors to either a higher or lower mean birth weight, and a consequent over or under categorization of low birth weight based on the 2500 g cutoff.

### Modeling Birth Weight as a Distribution to Improve High-Risk Infant Identification

In the late 1960s researchers began to use more sophisticated modeling techniques to describe birth weight. The aims were to improve discrimination of at risk births within and between populations relative to the 2500 g standard. In 1968 Brimblecombe proposed two Gaussian distributions to describe birth weight (Brimblecombe and Ashford 1968). He wrote that the majority of births comprise the primary Gaussian distribution and a minority of high-risk births to a second Gaussian distribution centered at the lower tail of the primary distribution. This was one of the earliest papers where the classification of low birth weight was based on distributional components rather than categorical groupings.

Adams, in 1969, developed a probability model, based on Native American infants, to describe the chance that an infant in the lower tail of the probability distribution was part of the normal or “deviant” distribution (Adams et al. 1968). The model proposed that as birth weight decreased the probability of belonging to the deviant population increased. Pethybridge noted five components to describe the two distributions (means, standard deviations, and proportion in the secondary distribution) (Fryer and Robertson 1972; Pethybridge et al. 1974). However, he used a simplified 3-component model using the mean and standard deviation of the primary distribution and a cutoff of 2000 g leaving 2 % of births in the secondary distribution. Sansing developed a conditional survival probability model and applied it to previously published datasets (Sansing and Chinnici 1976). This model identified the lower discriminating birth weight where the probability of dying was higher than that of surviving. Rooth, noting the differences in mean birth weights between populations, argued that the universal 2500 g was inadequate (Rooth 1980). He proposed using a cutoff of weights less than two standard deviations below the local population mean. The determination of whether differences between populations were due to genetic, environmental, or a combination of factors was not explored. The goal was to have a measure of low birth weight that better predicted neonatal mortality risk.

In 1983 Wilcox and Russell proposed an improved model in a series of papers that included three parameters: mean and standard deviation of the primary distribution and the proportion of all births in the residual distribution (Wilcox and Russell 1983a, b, 1986). They argued that their model accounted for the paradox that while males are heavier at birth they have higher neonatal mortality, which argues against the causal role of birth weight in mortality. If the cutoff was based on a population-specific distribution, females, for example, would have a lower low birth weight cutoff, leading to a smaller percentage categorized as low birth weight. Therefore, the proxy of weight for mortality risk would be more appropriate since females have lower neonatal mortality than males and would have a lower proportion categorized as low birth weight (or high risk for mortality). This finding, categorized as Simpson’s paradox, would help drive the need for population-specific curves. The final paper in their series expanded on birth weight and perinatal mortality (Wilcox and Russell 1986). More explicit in this paper is the description of two sources of neonatal mortality, which may confound comparisons between populations using previous methods. One source of mortality comes from excess numbers of births in the residual distribution; the second source is an excess number of deaths across the entire distribution. They described the combination of a weight-specific mortality curve and the frequency distribution of birth weight to compare mortality between populations to avoid bias that occurs with other standardization methods.

The Wilcox-Russell birth weight standardization process was applied to examine the different contributions of gestational age and birth weight to perinatal mortality (Wilcox 1993). Wilcox applied this model to the association of maternal smoking and birth weight with perinatal mortality (Wilcox 1993). Mothers who smoke have infants who have lower birth weight than infants of non-smoking mothers. Overall, smoking mothers have higher infant mortality. Yet, at every low birth weight category infants of mothers who smoked had lower mortality than non-smoking mothers. However, when the weights are standardized, smoking-exposed infants have higher mortality at every weight category. The comparison of the smoking and non-smoking birth weight distributions through standardization provided a clearer picture of the risk smoking posed to infants than comparison on an absolute scale. A second example used the exposure of high altitude comparing weight-specific mortality in Colorado versus the United States. Colorado and the US had comparable neonatal mortality; however, at low weights, Colorado infants had lower mortality than US infants in general. When standardized using a z-score, mortality was comparable across the weight spectrum. This demonstrated that Colorado infants had a shift in their birth weight curve without affecting overall mortality.

### Continued Controversy on the Utility of Birth Weight

Despite the arguments in the literature by Wilcox, researchers and the World Health Organization continued to compare birth weights between populations and include reduction of low birth weight as a major goal despite evidence against its causal role in neonatal mortality, independent of its component etiologies of preterm and intrauterine growth restriction (Wardlaw 2004). A footnote in the report stated, “The WHO definition of low birthweight serves for comparative health statistics and is not appropriate for clinical care.”

### Searching for a Causal Model

According to Wilcox, birth weight is not in the causal pathway to neonatal mortality. There must be other confounding factor(s) associated with birth weight that also increase mortality risk. Increasing birth weight is a health target for many countries to reduce infant mortality. However, these efforts may be misguided if birth weight is not a causal factor, although it may be a reasonable surrogate measure of a successful policy. More recent work has focused on the epidemiology of preterm and SGA as these are causal factors in neonatal morbidity and mortality (Katz et al. 2013; Lawn et al. 2010; Lee et al. 2013). Furthermore, it has been shown that infants who are term SGA but not low birth weight are at increased mortality risk compared with AGA infants (Katz et al. 2013). The timing and causes of death may also differ for preterm versus SGA infants, a higher proportion of preterm deaths occurring early in the neonatal period whereas SGA deaths tend to occur later in the neonatal period and through infancy (Katz et al. 2013).

### Standard, Population-Specific, Custom, and Ideal Birth Weight for Gestational Age Curves

While WHO provides one low birth weight standard for the world it acknowledges “it has also become increasingly evident that the cut-off value of 2500 g may not be appropriate for all settings. Some countries, with high incidence of low birthweight do not necessarily have high mortality rates, as for example in Sri Lanka” (Wardlaw 2004). In addition, there was a greater appreciation for the varying maternal risk factors causally linked to SGA versus preterm which should drive different interventions to reduce these adverse pregnancy outcomes (Kramer 1987). Starting in the 1990s researchers began to develop population specific and custom birth weight curves to help demarcate normal and poor fetal growth in a specific setting or individual fetus. Population-based standards define SGA as infants below a certain percentile (e.g. <10th) or below 2 standard deviations of the reference population mean for birth weight and gestational age. Custom curves produce an ideal fetal growth trajectory for each pregnancy based on maternal characteristics (Hadlock et al. 1991).

Gardosi developed a custom fetal growth weight standard that could be adjusted based on maternal factors and fetal sex (Gardosi et al. 1995; Hadlock et al. 1991). A weight-for-gestational age function could be used clinically to monitor SGA given a pregnancy’s specific characteristics (Figueras and Gardosi 2009). The model was applied in a variety of settings including New Zealand (McCowan et al. 2004), Spain (Figueras et al. 2007, 2008), the United States(Gardosi and Francis 2009), France (Ego et al. 2006) and Sweden (Clausson et al. 2001). Comparisons between these two methods in identifying high-risk infants were made. In a Swedish population, the custom model increased identification of high-risk infants compared to the population-based model (Clausson et al. 2001). Others reexamined the Gardosi model and questioned its validity given it was based on fetal weight, which differs from birth weight (Zhang et al. 2007).

Despite evidence against Gardosi’s model its momentum continued with the British Royal College of Obstetricians and Gynaecologists and other professional associations supporting customized birth weight percentiles (Hutcheon et al. 2011). A seminal *Lancet* paper described a global reference for fetal-weight and birth weight percentiles (Mikolajczyk et al. 2011). Three methods were compared using data from the 2004–2008 Global Survey on Maternal and Perinatal Health: (1) standard fetal growth curve, (2) country-specific fetal growth curve, and (3) Gardosi’s custom fetal growth curves with adjustment factors for individualization. They found the latter two methods comparable. In a later study, the global reference technique was applied to a US population-based cohort, which improved infant mortality prediction compared to a standard reference (Ding et al. 2013).

INTERGROWTH-21 sought to develop a universal global standard for normal fetal growth (Uauy et al. 2013). Fetal growth was prospectively monitored in healthy pregnancies under ideal conditions. This included a cohort that followed fetal weights longitudinally in healthy pregnancies in 8 countries, as well as a larger cross-sectional analysis of birth weights by gestational age that have been used to create a new birth weight for gestational age reference population. Previous reference populations have mostly used data from all pregnancies regardless of the environmental conditions surrounding the pregnancies. Initial results were recently published on birth weight, length and fetal growth across eight geographic regions (Papageorghiou et al. 2014; Villar et al. 2014, 2016). The variation in birth length was minimal among healthy women. This implies that genetic contributions to fetal growth play a minimal role compared to environmental factors. Now that optimal fetal growth curves are available, they are increasingly being adopted as a global standard against which to identify SGA. Research to identify the mortality and morbidity risks associated with varying deviations from this ideal growth will be helpful in clinical care and public health practice.

## Summary

Low birth weight continues to be an accessible measure of mortality risk in resource limited settings (World Health Organization 2012). However, increasing access to early ultrasound measurement of gestational age in low resource settings should lead clinicians and public health professionals towards differentiating SGA from preterm. Such distinctions would allow public health practitioners to better target and track the impact of interventions to prevent these adverse pregnancy outcomes. The new fetal growth standards will likely help move researchers and public health practitioners away from low birth weight and towards the more biologically useful descriptions of growth restriction and preterm, although birth weight will continue to be a convenient surrogate for preterm and SGA births, and a strong predictor of early mortality.

## References

[CR1] Adams MS, MacLean CJ, Niswander JD (1968). Discrimination between deviant and ordinary low birth weight: American Indian infants. Growth.

[CR2] Anderson NA, Lyon RA (1939). Causes of prematurity. American Journal of Diseases of Children.

[CR3] Ballantyne JW (1902). The problem of the premature infant. British Medical Journal.

[CR4] Battaglia FC, Frazier TM, Hellegers AE (1966). Birth weight, gestational age, and pregnancy outcome, with special reference to high birth weight-low gestational age infant. Pediatrics.

[CR5] Battaglia FC, Lubchenco LO (1967). A practical classification of newborn infants by weight and gestational age. The Journal of Pediatrics.

[CR6] Brimblecombe FS, Ashford JR (1968). Significance of low birth weight in perinatal mortality. A study of variations within England and Wales. British Journal of Preventive & Social Medicine.

[CR7] Budin, P. (1907). The nursling: The feeding and hygiene of premature and full-term infants. (W. J. Maloney, Trans.). London: The Caxton Publishing Company. doi:10.1016/S0033-3506(06)80187-3

[CR8] Capper A (1928). The fate and development of the immature and of the premature child. American Journal of Diseases of Children.

[CR9] Chase HC (1969). Infant mortality and weight at birth: 1960 United States birth cohort. American Journal of Public Health and the Nation’s Health.

[CR10] Clausson B, Gardosi J, Francis A, Cnattingius S (2001). Perinatal outcome in SGA births defined by customised versus population-based birthweight standards. BJOG: An International Journal of Obstetrics & Gynaecology.

[CR11] Clifford SH (1934). A consideration of the obstetrical management of premature labor. The New England Journal of Medicine.

[CR12] Clifford SH (1934). Reduction of premature infant mortality through determination of fetal size in utero. Journal of the American Medical Association.

[CR13] Committee to Study the Prevention of Low Birthweight, D. O. H. P., & Prevention, D. (1985). *Preventing low birthweight*. Washington: The National Academies Press. 25009946

[CR14] Ding G, Tian Y, Zhang Y, Pang Y, Zhang J, Zhang J (2013). Application of A global reference for fetal-weight and birthweight percentiles in predicting infant mortality. BJOG: An International Journal of Obstetrics & Gynaecology.

[CR15] Ego A, Subtil D, Grange G, Thiebaugeorges O, Senat M-V, Vayssiere C, Zeitlin J (2006). Customized versus population-based birth weight standards for identifying growth restricted infants: A French multicenter study. American Journal of Obstetrics and Gynecology.

[CR16] Erhardt CL, Joshi GB, Nelson FG, Kroll BH, Weiner L (1964). Influence of weight and gestation on perinatal and neonatal mortality by ethnic group. American Journal of Public Health and the Nation’s Health.

[CR17] Expert Group on Prematurity. (1950). *Expert Group on Prematurity* (No. 27, 6 ed.). Geneva: World Health Organization. 14782513

[CR18] Figueras F, Figueras J, Meler E, Eixarch E, Coll O, Gratacos E (2007). Customised birthweight standards accurately predict perinatal morbidity. Archives of Disease in Childhood. Fetal and Neonatal Edition.

[CR19] Figueras F, Gardosi J (2009). Should we customize fetal growth standards?. Fetal Diagnosis and Therapy.

[CR20] Figueras F, Meler E, Iraola A, Eixarch E, Coll O, Figueras J (2008). Customized birthweight standards for a Spanish population. European Journal of Obstetrics & Gynecology and Reproductive Biology.

[CR21] Fryer JG, Robertson CA (1972). A comparison of some methods for estimating mixed normal distributions. Biometrika.

[CR22] Gardosi J, Francis A (2009). A customized standard to assess fetal growth in a US population. American Journal of Obstetrics and Gynecology.

[CR23] Gardosi J, Mongelli M, Wilcox M, Chang A (1995). An adjustable fetal weight standard. Ultrasound in Obstetrics & Gynecology: The Official Journal of the International Society of Ultrasound in Obstetrics and Gynecology.

[CR24] Gruenwald P (1964). Infants of low birth weight among 5,000 deliveries. Pediatrics.

[CR25] Hadlock FP, Harrist RB, Martinez-Poyer J (1991). In utero analysis of fetal growth: A sonographic weight standard. Radiology.

[CR26] Hess JH, Chamberlin IM (1927). Premature infants. American Journal of Diseases of Children.

[CR27] Holt LE, Babbit EC (1915). Institutional mortality of the new-born. Journal of the American Medical Association.

[CR28] Hutcheon JA, Walker M, Platt RW (2011). Assessing the value of customized birth weight percentiles. Weekly Epidemiological Record.

[CR29] Karn MN, Penrose LS (1951). Birth weight and gestation time in relation to maternal age, parity and infant survival. Annals of Eugenics.

[CR30] Katz J, Lee AC, Kozuki N, Lawn JE, Cousens S, Blencowe H (2013). Mortality risk in preterm and small-for-gestational-age infants in low-income and middle-income countries: A pooled country analysis. The Lancet.

[CR31] Koch LA, Weymuller CA, James E (1948). Reduction of mortality from premature birth. Journal of the American Medical Association.

[CR32] Kramer MS (1987). Determinants of low birth weight: Methodological assessment and meta-analysis. Bulletin of the World Health Organization.

[CR33] Lawn JE, Gravett MG, Nunes TM, Rubens CE, Stanton C (2010). Global report on preterm birth and stillbirth (1 of 7): Definitions, description of the burden and opportunities to improve data. BMC Pregnancy and Childbirth.

[CR34] Lee AC, Katz J, Blencowe H, Cousens S, Kozuki N, Vogel JP (2013). National and regional estimates of term and preterm babies born small for gestational age in 138 low-income and middle-income countries in 2010. The Lancet Global Health.

[CR35] Lubchenco, L. O. (1976). Classification of high risk infants by birth weight and gestational age: an overview. In A. Schaffer (Ed.), *Major Problems in Clinical Pediatrics* (Vol. 14, pp. 1–8). W.B. Saunders Company. 979340

[CR36] Lubchenco LO, Hansman C, Dressler M, Boyd E (1963). Intrauterine growth as estimated from liveborn birth-weight data at 24 to 42 weeks of gestation. Pediatrics.

[CR37] Lubchenco LO, Searls DT, Brazie JV (1972). Neonatal mortality rate: Relationship to birth weight and gestational age. The Journal of Pediatrics.

[CR38] McCormick MC (1985). The contribution of low birth weight to infant mortality and childhood morbidity. The New England Journal of Medicine.

[CR39] McCowan L, Stewart AW, Francis A, Gardosi J (2004). A customised birthweight centile calculator developed for a New Zealand population. The Australian & New Zealand journal of obstetrics & gynaecology.

[CR40] Mikolajczyk RT, Zhang J, Betran AP, Souza JP, Mori R, Gülmezoglu AM, Merialdi M (2011). A global reference for fetal-weight and birthweight percentiles. Lancet.

[CR41] Optiz H (1914). Über Wachstum und Entwicklung untergewichtiger ausgetragener Neugeborener. Monatsschrift Kinderheilkunde.

[CR42] Papageorghiou AT, Ohuma EO, Altman DG, Todros T, Ismail LC, Lambert A (2014). International standards for fetal growth based on serial ultrasound measurements: The Fetal Growth Longitudinal Study of the INTERGROWTH-21st Project. The Lancet.

[CR43] Pearce NO (1919). Review of recent literature on the new-born. American Journal of Diseases of Children.

[CR44] Peckham CH (1938). Statistical studies on prematurity. The Journal of Pediatrics.

[CR45] Pethybridge RJ, Ashford JR, Fryer JG (1974). Some features of the distribution of birthweight of human infants. British Journal of Preventive & Social Medicine.

[CR46] Puffer, R. R., & Serrano, C. V. (1973). Patterns of mortality in childhood: Report of the Inter-American Investigation of Mortality in Childhood. *PAHO Scientific Publication*, 262 983115

[CR47] Record RG, Gibson JR, McKeown T (1952). Foetal and infant mortality in multiple pregnancy. BJOG: An International Journal of Obstetrics & Gynaecology.

[CR48] Rooth G (1980). Low birthweight revised. The Lancet.

[CR50] Sansing RC, Chinnici JP (1976). Optimal and discriminating birth weights in human populations. Annals of Human Genetics.

[CR51] Saugstad LF (1981). Weight of all births and infant mortality. Journal of Epidemiology and Community Health.

[CR52] Schlesinger ER, Allaway N (1955). The combined effect of birth weight and length of gestation on neonatal mortality among single premature births. Pediatrics.

[CR53] Schwarz H, Kohn JL (1921). The infant of low birth weight; it’s growth and development. American Journal of Diseases of Children.

[CR54] Shaw LA, Hopkins FR (1931). the respiration of premature infants. American Journal of Diseases of Children.

[CR55] Silverman WA, Lecey JF, Beard A, Brown AK, CornBlath M, Grossman M (1967). Committee on Fetus and newborn: Nomenclature for duration of gestation. Birth Weight and Intra-Uterine Growth.

[CR56] Steiner M, Pomerance W (1950). Studies on prematurity II. Influence of Fetal Maturity on Fatality Rate.

[CR57] Taback M (1951). Birth weight and length of gestation with relation to prematurity. Journal of the American Medical Association.

[CR58] Talbot FB, Sisson WR, Moriarty ME, Dalrymple AJ (1923). The basal metabolism of prematurity. American Journal of Diseases of Children.

[CR59] Taylor R (1918). Treatment of prematurity. Journal of the American Medical Association.

[CR60] Uauy R, Casanello P, Krause B, Kuzanovic JP, Corvalan C (2013). Conceptual basis for prescriptive growth standards from conception to early childhood: Present and future. BJOG: An International Journal of Obstetrics & Gynaecology.

[CR61] van den Berg BJ, Yerushalmy J (1966). The relationship of the rate of intrauterine growth of infants of low birth weight to mortality, morbidity, and congenital anomalies. The Journal of Pediatrics.

[CR62] Villar J, Giuliani F, Fenton TR, Ohuma EO, Ismail LC, Kennedy SH (2016). INTERGROWTH-21st very preterm size at birth reference charts. The Lancet.

[CR63] Villar J, Papageorghiou AT, Pang R, Ohuma EO, Ismail LC, Barros FC (2014). The likeness of fetal growth and newborn size across non-isolated populations in the INTERGROWTH-21st Project: The Fetal Growth Longitudinal Study and Newborn Cross-Sectional Study. The Lancet Diabetes & Endocrinology.

[CR64] Wardlaw, T. M. (2004). *Low birthweight: Country, regional and global estimates*. United Nations Children’s Fund and World Health Organization.

[CR65] Wilcox AJ (1993). Birth weight and perinatal mortality: The effect of maternal smoking. Weekly Epidemiological Record.

[CR66] Wilcox AJ (2001). On the importance—and the unimportance—of birthweight. International Journal of Epidemiology.

[CR67] Wilcox AJ, Russell IT (1983). Birthweight and perinatal mortality: I. On the frequency distribution of birthweight. International Journal of Epidemiology.

[CR68] Wilcox AJ, Russell IT (1983). Perinatal mortality: Standardizing for birthweight is biased. Weekly Epidemiological Record.

[CR69] Wilcox AJ, Russell IT (1986). Birthweight and perinatal mortality: III. Towards a new method of analysis. International Journal of Epidemiology.

[CR70] Williams RL, Creasy RK, Cunningham GC, Hawes WE, Norris FD, Tashiro M (1982). Fetal growth and perinatal viability in California. Obstetrics and Gynecology.

[CR71] World Health Organization. (2012). *WHA65.6 Comprehensive implementation plan on maternal, infant and young child nutrition*. Retrieved from http://www.who.int/nutrition/topics/wha_65_6/en/10.3945/an.114.007781 PMC4288273 25593153

[CR72] Ylppö A (1919). Pathologisch-anatomische Studien bei Frühgeborenen Makroskopische und mikroskopische Untersuchungen mit Hinweisen auf die Klinik und mit besonderer Berücksichtigung der Hämorrhagien. European Journal of Pediatrics.

[CR73] Zhang X, Platt RW, Cnattingius S, Joseph KS, Kramer MS (2007). The use of customised versus population-based birthweight standards in predicting perinatal mortality. BJOG: An International Journal of Obstetrics & Gynaecology.

